# Effects of patient factors on inpatient mortality after complex liver, pancreatic and gastric resections

**DOI:** 10.1002/bjs5.33

**Published:** 2018-03-15

**Authors:** V. M. Zaydfudim, G. J. Stukenborg

**Affiliations:** ^1^ Department of Surgery University of Virginia School of Medicine Charlottesville, Virginia USA; ^2^ Department of Public Health Sciences University of Virginia School of Medicine Charlottesville, Virginia USA; ^3^ Department of Surgical Outcomes Research Center University of Virginia School of Medicine Charlottesville, Virginia USA

## Abstract

**Background:**

There is mixed evidence that patients who receive care in hospitals with a low case volume for complex gastrointestinal and hepatobiliary operations have an increased risk of inpatient death.

**Methods:**

A retrospective cohort study was performed of patients who had complex gastrointestinal and hepatobiliary operations in the Healthcare Cost and Utilization Project 2012 National Inpatient Sample. Multivariable weighted hierarchical generalized linear models were used to test the relationship between hospital case volume and probability of inpatient death, with detailed adjustments for the concurrent effects of differences in associated patient co‐morbidities.

**Results:**

A total of 8260 pancreaticoduodenectomies, 2750 major hepatectomies and 3250 total gastrectomies were identified. Inpatient death occurred in 3·6 per cent of patients after pancreaticoduodenectomy, 4·9 per cent after major hepatectomy and 4·6 per cent after total gastrectomy. Mean hospital case volume was 50·6 (median 40) for pancreaticoduodenectomy, 23·6 (median 15) for major hepatectomy, 15·1 (median 10) for total gastrectomy and 70·2 (median 50) for any of the three operations. Hospital case volume was not a statistically significant predictor of mortality after any operation (all P ≥ 0·188). Patient characteristics including age and co‐morbidity were highly significant predictors of mortality (P < 0·001). No significant improvements in model performance were obtained by adding hospital case volume to any model that already included adjustments for patient‐level differences in age and co‐morbid disease, for any functional format (P ≥ 0·146 for all C statistic differences from baseline).

**Conclusion:**

Patient co‐morbidity, not hospital case volume, was associated with significant differences in inpatient mortality following complex gastric, pancreatic and hepatobiliary resections.

## Introduction

The relationship between hospital case volume for complex operations and the risk of inpatient death has been a topic of research interest and healthcare policy debate for decades. A landmark 2002 study^1^ found that low hospital case volume for pancreatic resection was associated with a significantly increased risk of 30‐day mortality. Other studies^2^, ^3^, ^4^ have demonstrated significant associations between volume and mortality among patients selected for complex gastrointestinal (GI) and hepatopancreatobiliary (HPB) resections. Although other studies^5^ ^6^ have indicated that the relationship between case volume and mortality risk is not significant, there is substantial variation in the statistical methods used to assess the relationship between hospital volume and outcomes^7^ ^8^. Many studies have used statistical methods that do not account for the hierarchical structure of the data when analysing differences in outcomes among patients clustered within hospitals^9^, ^10^, ^11^, ^12^. Studies also vary in how they account for baseline differences between patients when determining the independent effect of hospital case volume^13^.

Meaningful reductions in mortality and changes in standards of practice have occurred over recent decades. Perioperative mortality for major cancer resections has declined significantly^14^. Provider, institutional and organizational quality improvement efforts have altered the patient care paradigm, with more patients receiving complex cancer surgical care at specialist centres^15^. Healthcare delivery system consolidation has reduced the number of hospitals offering complex GI and HPB resections^16^ ^17^.

The use of heterogeneous statistical methods, differences in study populations, changes in practice and the overall reduction in short‐term mortality limit the applicability of evidence from older studies to the present era. This study examined the relationship between hospital case volume and inpatient mortality for patients after three specific complex gastrointestinal and hepatobiliary operations using updated statistical methods and population data, with detailed adjustment for the confounding effects of patient‐level differences in co‐morbid disease.

## Methods

Data were obtained from the Healthcare Cost and Utilization Project (HCUP) 2012 National Inpatient Sample (NIS)^18^. The HCUP 2012 NIS approximates a 20 per cent stratified sample of hospital discharges, drawn from a sampling frame that covers 95 per cent of the population discharged from non‐federal hospitals in the USA. The ICD‐9‐CM code reported as the principal operation performed was used to identify all reported patients in the HCUP 2012 NIS data for radical pancreaticoduodenectomy (52·7), major hepatic lobectomy (50·3) and total gastrectomy (43·9, 43·91, or 43·99). Each case in the data set was weighted to reflect the hospital discharge sampling frame. Weighted cases were used for all analyses performed in this study.

Hospital case volume was measured as the total number of weighted cases reported by hospitals for each procedure, and for the collective total of all three procedures. Items recorded for each patient included the occurrence of inpatient death, type of healthcare insurance (Medicare, Medicaid, private, self‐pay, indigent, other payer) and age. Dichotomous indicators for 29 categories of co‐morbid disease were defined for each patient, based on ICD‐9‐CM diagnostic codes, reported as secondary diagnoses using classification software available from the US Agency for Healthcare Research and Quality^19^ ^20^. Weighted cases with missing data among the pertinent co‐variable or outcome measures were excluded. The overall missing value rates for most variables in HCUP 2012 NIS are less than 1 per cent^21^.

### Statistical analysis

Multivariable weighted hierarchical generalized linear models (HGLMs) were used to estimate the magnitude and statistical significance of the effect of hospital case volume on the probability of inpatient death, adjusted for the concurrent effects of selected patient‐level co‐variables^22^. The study data set included both hospital‐ and patient‐level measures, and was thus hierarchically structured, with records for individual patients stratified within hospitals. Within the data set, records for patients associated with the same hospital had identical hospital volume measures for each of the three procedures and for the collective total. Statistical models that incorporated predictive effects for both hospital‐ and patient‐level co‐variables had variance components and model error terms that were estimated appropriately within the context of HGLM formulation^9^. Multivariable weighted HGLMs were estimated separately for each of the three procedure‐defined study populations, and for the combined population. All models were formulated with *a priori* selection of model co‐variables.

The effect of hospital volume on patient mortality risk was assessed using linear, dichotomous and polynomial functional formats. A linear function was assessed using hospital case volume as a continuous variable. A dichotomous function was assessed using a categorical variable that grouped patients into hospitals with volumes either below or at or above the median volume. A non‐linear function was estimated using restricted cubic spline polynomials. Although segmenting continuous variables into categories is a common method for assessing non‐linearity, this practice eliminates much of the information available from the case volume measure^23^. Restricted cubic splines are piecewise polynomial functions that are fitted to the relationship between case volume and the estimated probability of mortality, with the ends of the functions restricted to be linear^24^, ^25^, ^26^. The cubic spline functions were estimated with knots set at the fifth, 50th and 95th percentiles, with the spline function tails restricted to be linear at the fifth and 95th percentiles of the volume distributions, for each measure.

The multivariable weighted HGLMs included separate intercept terms for each hospital. The effect of hospital case volume on the probability of inpatient death was adjusted for the concurrent effects of patient age and for categories of co‐morbid disease that occurred in 5 per cent or more of the study population. Rarely occurring categories of co‐morbid disease were excluded from the models to reduce the number parameter estimates needed for model convergence.

The statistical significance of each model co‐variable was assessed using type III tests of fixed effects (*F* test statistics), which reflect the proportion of the total model log‐likelihood independently explained by each model co‐variable. The statistical significance of the *F* test statistics obtained for each model co‐variable was assessed at the *P* < 0·001 threshold.

Nested models were used to assess the relative contribution of hospital case volume to the estimation of patient mortality risk, compared with the contribution made by patient‐level differences in age and co‐morbid disease burden. Each HGLM was compared with an otherwise identical model that excluded the hospital volume co‐variable. Nested model pairs were assessed for each of the three procedures and for the combined population, for each of the three functions representing the effect of volume on the probability of inpatient death.

The capacity of each model to discriminate between patients discharged alive or dead was measured using the C statistic^27^ ^28^. A C statistic of 0·5 indicates that the model provided no predictive discrimination, whereas a value of 1·0 indicates perfect discrimination. The statistical significance of the difference between the C statistic for each nested model pair was assessed at the *P* < 0·001 threshold^29^.

Results from the paired models were also compared using the integrated discrimination improvement (IDI) statistic to measure the effect of each volume measure on model reclassification^30^. Reclassification addresses the extent to which a model including hospital case volume correctly (or incorrectly) reclassifies patients as having died or survived compared with classification of the same patient using an otherwise identical model without the hospital volume co‐variable. The IDI statistic obtains a value of −100 per cent for the maximum possible decline in the overall sensitivity and specificity, 0 per cent for no difference, and 100 per cent for the maximum possible improvement. The significance of the difference between the IDI statistics for each nested model pair was also assessed at the *P* < 0·001 threshold.

Statistical significance was assessed at the *a priori* threshold value of *P* < 0·001 for each comparison made in order to account for the large size of the weighted sample data set and for the potential for multiple comparisons bias^31^. In total, 16 multivariable weighted HGLMs were developed in the available data. Data management and weighted HGLM analysis was conducted using SAS® version 9.4 (SAS Institute, Cary, North Carolina, USA). Graphics programming was conducted using R version 3.2.1 statistical software (R Foundation for Statistical Computing, Vienna, Austria).

## Results

The NIS 2012 HCUP database contained 7 296 968 hospital discharge records, selected from a weighted national sample of the total population of 146 million hospital discharges reported during 2012 for all US non‐federal short‐term general and other specialty hospitals. A total of 8260 pancreaticoduodenectomies, 2750 major hepatectomies and 3250 total gastrectomies were identified. Pancreaticoduodenectomy was reported by 432 hospitals, major hepatectomy by 253 and total gastrectomy by 381; 653 hospitals reported at least one patient for any of the three operations.

**Figure 1:**
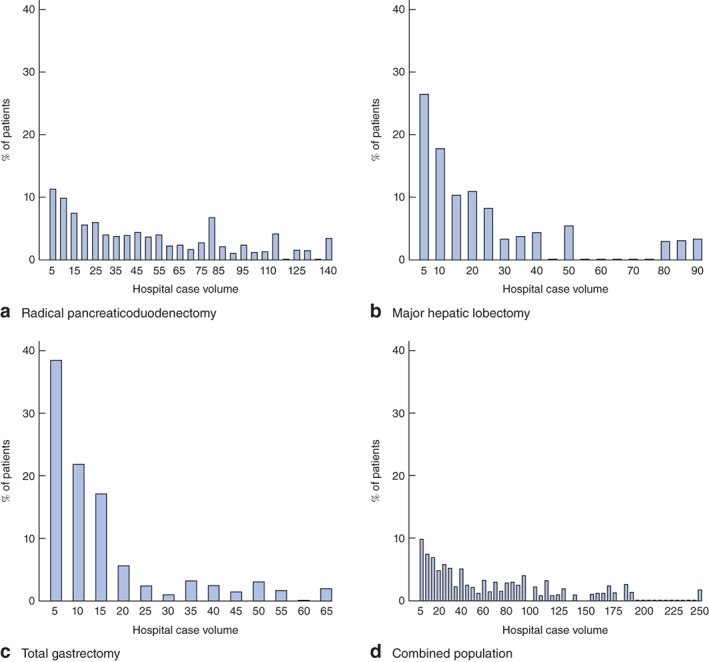
Frequency histograms of weighted hospital case volume distributions for **a** radical pancreaticoduodenectomy, **b** major hepatic lobectomy, **c** total gastrectomy and **d** the combined population

Weighted frequency distributions of hospital case volume for the three operations, individually and in combination are shown in *Fig*. 1. Mean hospital case volume was 50·6 (median 40; weighted range 5–150) for pancreaticoduodenectomy, 23·6 (15; 5–900) for major hepatectomy, 15·1 (10; 5–65) for total gastrectomy and 70·2 (50; 5–275) for any of the three operations. Case volumes greater than five were reported by 88·7 per cent of hospitals for pancreaticoduodenectomy, by 73·4 per cent for major hepatectomy, by 61·5 per cent for total gastrectomy and by 90·1 per cent for the three operations combined. More than ten cases were performed by 78·9 per cent of hospitals for pancreaticoduodenectomy, by 55·6 per cent for major hepatectomy, by 39·7 per cent for total gastrectomy and by 82·7 per cent for any of the three operations.

Multivariable weighted HGLMs were estimated with separate intercept terms for each hospital, and the effect of hospital case volume on the probability of inpatient death was adjusted for the concurrent effects of patient age and co‐morbid disease. The frequency of each patient characteristic in each of the three procedure‐specific study populations is shown in *Table* 1. Inpatient death occurred in 3·6 per cent of patients after pancreaticoduodenectomy, 4·9 per cent after major hepatectomy and 4·6 per cent after total gastrectomy.

**Table 1:** Characteristics of the study population

	Pancreaticoduodenectomy	Major hepatectomy	Total gastrectomy
Total no. of patients (weighted)	8260 (100)	2750 (100)	3250 (100)
Inpatient death	300 (3·6)	135 (4·9)	150 (4·6)
Healthcare funding	(*n* = 8205)	(*n* = 2745)	(*n* = 3245)
Medicare	4225 (51·5)	885 (32·2)	1540 (47·5)
Medicaid	430 (5·2)	245 (8·9)	295 (9·1)
Private insurance	3060 (37·3)	1375 (50·1)	1260 (38·8)
Self‐pay	210 (2·6)	125 (4·6)	45 (1·4)
Indigent	25 (0·3)	10 (0·4)	5 (0·2)
Other payer	255 (3·1)	105 (3·8)	100 (3·1)
Age (years)			
< 40	295 (3·6)	450 (16·4)	150 (4·6)
40–49	780 (9·4)	390 (14·2)	340 (10·5)
50–59	1795 (21·7)	660 (24·0)	860 (26·5)
60–69	2770 (33·5)	695 (25·3)	1015 (31·2)
70–79	2065 (25·0)	445 (16·2)	680 (20·9)
80–89	555 (6·7)	110 (4·0)	205 (6·3)
Co‐morbidity			
Acquired immune deficiency syndrome	10 (0·1)	5 (0·2)	0 (0)
Alcohol abuse/dependency	305 (3·7)	70 (2·5)	100 (3·1)
Chronic blood loss anaemia	105 (1·3)	40 (1·5)	125 (3·8)
Chronic pulmonary disease	1025 (12·4)	360 (13·1)	580 (17·8)
Coagulopathy	600 (7·3)	350 (12·7)	265 (8·2)
Congestive heart failure	220 (2·7)	65 (2·4)	170 (5·2)
Deficiency anaemia	1455 (17·6)	345 (12·5)	710 (21·8)
Depression	725 (8·8)	210 (7·6)	275 (8·5)
Diabetes, uncomplicated	2240 (27·1)	390 (14·2)	615 (18·9)
Diabetes with chronic complications	230 (2·8)	35 (1·3)	80 (2·5)
Drug abuse	110 (1·3)	40 (1·5)	20 (0·6)
Fluid and electrolyte disorders	3080 (37·3)	790 (28·7)	1180 (36·3)
Hypertension	4800 (58·1)	1205 (43·8)	1735 (53·4)
Hypothyroidism	870 (10·5)	175 (6·4)	315 (9·7)
Liver disease	270 (3·3)	380 (13·8)	85 (2·6)
Lymphoma	25 (0·3)	10 (0·4)	5 9 (0·2)
Metastatic cancer	2685 (32·5)	395 (14·4)	805 (24·8)
Obesity	985 (11·9)	265 (9·6)	305 (9·4)
Other neurological disorder	240 (2·9)	100 (3·6)	130 (4·0)
Paralysis	50 (0·6)	10 (0·4)	25 (0·8)
Peptic ulcer disease excluding bleeding	25 (0·3)	0 (0)	5 (0·2)
Peripheral vascular disorders	375 (4·5)	80 (2·9)	115 (3·5)
Psychosis	175 (2·1)	50 (1·8)	80 (2·5)
Pulmonary circulation disorder	210 (2·5)	70 (2·5)	125 (3·8)
Renal failure	350 (4·2)	120 (4·4)	210 (6·5)
Rheumatoid arthritis	175 (2·1)	25 (0·9)	55 (1·7)
Solid tumour without metastasis	355 (4·3)	390 (14·2)	120 (3·7)
Valvular disease	310 (3·8)	100 (3·6)	105 (3·2)
Weight loss	2020 (24·5)	255 (9·3)	770 (23·7)

Hospital case volume was not a statistically significant predictor of mortality in any study population, for any functional format (all *P* ≥ 0·094). *Table* 2 lists the effect and statistical significance of hospital case volume on inpatient mortality risk for each study population, for each functional format, adjusted for the concurrent effects of patient‐level co‐variables. Co‐variable effects are expressed as odds ratios (ORs), with 95 per cent confidence intervals, for effects referent to selected groups. ORs for the effect of hospital case volume measured by the linear function are represented in increments of 5‐, 10‐ and 50‐unit increases. For hospital case volume represented as a dichotomous function, ORs are expressed with reference to median volume. For example, hospital case volume for pancreaticoduodenectomy was not a statistically significant predictor of mortality, whether measured as a linear (*P* = 0·858), dichotomous (*P* = 0·815) or non‐linear spline function (components *P* = 0·419–0·622). The difference in the odds of death associated with a 50‐unit increase in pancreaticoduodenectomy case volume (OR 0·85, 95 per cent c.i. 0·16 to 4·66) was not statistically significant.

**Table 2:** Statistical significance of hospital case volume, odds of inpatient death, and effect of hospital case volume on model performance

	Case volume			Model C statistic	*P* value for C statistic difference from baseline	IDI statistic	*P* value for IDI statistic difference from baseline
	*F* test statistic	*P* value for *F* test statistic	Odds ratio				
Pancreaticoduodenectomy							
Baseline: excludes case volume	n.a.	n.a.	n.a.	0·80	n.a.	n.a.	n.a.
Linear: volume continuous	0·03	0·858					
Unit increase of 5 cases			0·98 (0·83, 1·17)				
Unit increase of 10 cases			0·97 (0·69, 1·36)				
Unit increase of 50 cases			0·85 (0·16, 4·66)	0·81	0·684	< 0·001	0·769
Dichotomous: volume split at median	0·05	0·815					
< median volume			1·32 (0·12, 14·06)				
≥ median volume			0·75 (0·07, 7·99)	0·81	1·000	< 0·001	0·714
Non‐linear: volume spline function							
Linear only	0·65	0·419					
Non‐linear part 1	0·33	0·567					
Non‐linear part 2	0·24	0·622		0·81	1·000	< 0·001	0·371
Major hepatectomy							
Baseline: excludes case volume	n.a.	n.a.	n.a.	0·78	n.a.	n.a.	n.a.
Linear: volume continuous	0·05	0·828					
Unit increase of 5 cases			0·86 (0·22, 3·41)				
Unit increase of 10 cases			0·81 (0·05, 12·87)				
Unit increase of 20 cases			0·54 (< 0·01, 135·29)	0·81	1·000	< 0·001	0·878
Dichotomous: volume split at median	0·09	0·765					
< median volume			1·00 (0·34, 2·92)				
≥ median volume			0·30 (< 0·01, 814·88)	0·82	1·000	< 0·001	0·643
Non‐linear: volume spline function							
Linear only	< 0·01	0·988					
Non‐linear part 1	0·08	0·772					
Non‐linear part 2	0·10	0·747		0·82	1·000	< 0·001	0·864
Total gastrectomy							
Baseline: excludes case volume	n.a.	n.a.	n.a.	0·79	n.a.	n.a.	n.a.
Linear: volume continuous	0·54	0·464					
Unit increase of 5 cases			0·49 (0·07, 3·32)				
Unit increase of 10 cases			0·24 (< 0·01, 11·04)				
Unit increase of 20 cases			0·06 (< 0·01, 121·34)	0·80	0·313	< 0·001	0·895
Dichotomous: volume split at median	1·74	0·188					
< median volume			30·15 (0·17, > 999·99)				
≥ median volume			0·03 (< 0·01, 5·43)	0·81	0·146	< 0·001	0·500
Non‐linear: volume spline function							
Linear only	1·35	0·247					
Non‐linear part 1	< 0·01	> 0·999					
Non‐linear part 2	< 0·01	> 0·999		0·80	0·212	< 0·001	0·523
Combined cases							
Baseline: excludes case volume	n.a.	n.a.	n.a.	0·80	n.a.	n.a.	n.a.
Linear: volume continuous	0·09	0·769					
Unit increase of 5 cases			0·98 (0·89, 1·08)				
Unit increase of 10 cases			0·97 (0·80, 1·17)				
Unit increase of 50 cases			0·86 (0·33, 2·23)	0·81	0·302	< 0·001	0·975
Dichotomous: volume split at median	0·20	0·658					
< median volume			1·48 (0·25, 8·73)				
≥ median volume			0·67 (0·11, 3·93)	0·81	0·422	< 0·001	0·116
Non‐linear: volume spline function							
Linear only	2·80	0·094					
Non‐linear part 1	1·38	0·240					
Non‐linear part 2	1·04	0·307		0·81	0·854	< 0·001	0·783

Values in parentheses are 95 per cent confidence intervals. n.a., not applicable.

**Figure 2:**
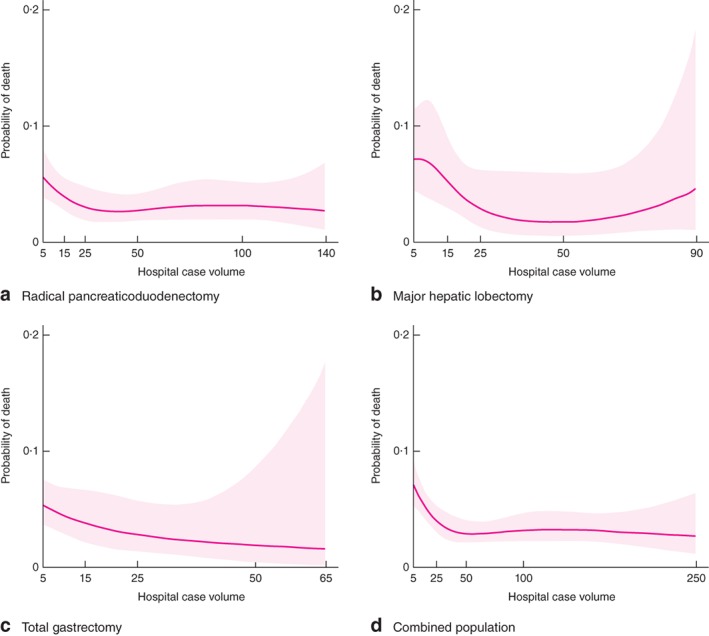
Restricted cubic spline functions and their associated 95 per cent confidence intervals for the relationship between probability of death and hospital case volume for **a** radical pancreaticoduodenectomy, **b** major hepatic lobectomy, **c** total gastrectomy and **d** the combined population

Restricted cubic spline functions estimated for the three study populations and for the combined population are shown in *Fig*. 2. Plotted functions illustrate that the fitted relationships were not sharply curved for any study population for probability of death in the range 0·0–0·1, and that there was little potential for statistically significant non‐linearity in the probability of death over the range of values for hospital case volume.

The nested model comparisons demonstrated that no significant improvements in model performance were obtained by adding hospital case volume to any model already including adjustments for patient‐level differences in age and co‐morbid disease, for any functional format (*Table* 2). For example, among patients with pancreaticoduodenectomy, a baseline model including only adjustments for age and co‐morbid disease yielded a C statistic of 0·80, indicating that the model adequately discriminated between surviving and deceased cases. Adding the effect of hospital case volume measured as a linear function improved the C statistic to 0·81, a slight improvement that was not statistically significant (*P* = 0·684). The IDI statistic obtained for this nested model comparison indicated that a less than 0·001 per cent increase in overall sensitivity and specificity was obtained by adding hospital case volume to the patient‐level co‐variables. This increase was not statistically significant (*P* = 0·769). Each of the nested model comparisons demonstrated that no significant improvement in model discrimination was obtained by adding hospital case volume to a baseline model adjusting for patient age and co‐morbidity.

In contrast to hospital case volume, many patient‐level characteristics were highly significant predictors of mortality (*P* < 0·001). The statistical significance and adjusted odds of inpatient death associated with each patient‐level co‐variable included in the multivariable weighted HGLMs with a linear effect for hospital volume are shown in *Table* 3. For example, in patients with pancreaticoduodenectomy, age (*P* < 0·001) was associated with a threefold increase in the odds of death per 10‐year increase in age (OR 3·09, 95 per cent c.i. 2·41 to 3·96). Patients with fluid and electrolyte disorders as defined by appropriate ICD‐9‐CM codes had a 12‐fold increased risk of death (OR 12·39, 7·12 to 21·57). Approximately equivalent test statistics and estimated ORs were obtained for each of the patient‐level co‐variables in the models where the effect of hospital case volume was represented using dichotomous or spline function formats.

**Table 3:** Statistical significance of patient‐level co‐variables and odds of inpatient death

	*F* test statistic	*P* value for *F* test statistic	Odds ratio
Pancreaticoduodenectomy			
Hospital case volume (unit increase of 50 cases)	0·03	0·858	0·85 (0·16, 4·66)
Age (unit increase of 10 years)	79·63	< 0·001	3·09 (2·41. 3·96)
Fluid and electrolyte disorders	79·32	< 0·001	12·39 (7·12, 21·57)
Coagulopathy	75·81	< 0·001	12·90 (7·25, 22·94)
Deficiency anaemia	32·20	< 0·001	0·13 (0·07, 0·26)
Peripheral vascular disorder	24·01	< 0·001	6·61 (3·10, 14·07)
Diabetes, uncomplicated	20·63	< 0·001	0·29 (0·17, 0·50)
Depression	14·56	0·001	3·31 (1·79, 6·11)
Obesity	11·38	0·001	3·22 (1·63, 6·36)
Hypertension	6·85	0·009	0·57 (0·38, 0·87)
Chronic pulmonary disease	6·73	0·009	0·40 (0·20, 0·80)
Weight loss	5·66	0·017	0·54 (0·32, 0·90)
Hypothyroidism	4·54	0·033	0·51 (0·27, 0·95)
Metastatic cancer	0·67	0·413	0·81 (0·48, 1·36)
Major hepatectomy			
Hospital case volume (unit increase of 50 cases)	0·05	0·828	0·54 (< 0·01, 135·29)
Age (unit increase of 10 years)	20·60	< 0·001	9·30 (3·54, 24·46)
Diabetes, uncomplicated	25·97	< 0·001	< 0·01 (< 0·01, < 0·01)
Coagulopathy	23·74	< 0·001	79·31 (13·55, 464·11)
Hypertension	19·12	< 0·001	0·01 (0·00, 0·09)
Solid tumour without metastasis	18·30	< 0·001	264·50 (20·32, > 999·99)
Liver disease	17·84	< 0·001	201·84 (17·02, > 999·99)
Fluid and electrolyte disorders	14·29	< 0·001	21·95 (4·39, 109·66)
Chronic pulmonary disease	2·36	0·125	< 0·01 (< 0·01, 8·73)
Metastatic cancer	1·24	0·266	0·21 (0·01, 3·29)
Deficiency anaemia	0·87	0·353	0·20 (0·01, 5·97)
Total gastrectomy			
Hospital case volume (unit increase of 50 cases)	0·54	0·464	0·06 (< 0·01, 121·34)
Age (unit increase of 10 years)	16·49	< 0·001	4·43 (2·15, 9·11)
Hypothyroidism	25·19	< 0·001	384·40 (37·20, > 999·99)
Fluid and electrolyte disorders	17·04	< 0·001	29·43 (5·86. 147·74)
Deficiency anaemia	6·78	0·009	0·05 (0·01, 0·49)
Weight loss	6·58	0·011	21·17 (2·03, 220·72)
Hypertension	5·06	0·025	0·25 (0·07, 0·84)
Diabetes, uncomplicated	4·70	0·031	4·28 (1·14, 16·04)
Obesity	3·48	0·063	7·21 (0·90, 57·97)
Chronic pulmonary disease	3·09	0·079	5·51 (0·82, 37·30)
Metastatic cancer	0·87	0·351	0·06 (< 0·01, 23·83)
Combined cases			
Hospital case volume (unit increase of 50 cases)	0·09	0·769	0·86 (0·33, 2·23)
Procedure group	5·10	0·006	
Pancreaticoduodenectomy			1·00 (reference)
Major hepatectomy			1·65 (1·14, 2·38)
Total gastrectomy			0·81 (0·55, 1·20)
Age (unit increase of 10 years)	119·42	< 0·001	2·23 (1·93, 2·57)
Coagulopathy	194·26	< 0·001	11·30 (8·03, 15·90)
Fluid and electrolyte disorders	86·16	< 0·001	4·01 (2·99, 5·38)
Deficiency anaemia	29·91	< 0·001	0·29 (0·18, 0·45)
Liver disease	23·77	< 0·001	3·23 (2·01, 5·19)
Hypertension	18·98	< 0·001	0·53 (0·40, 0·71)
Diabetes	10·00	0·002	0·57 (0·40, 0·80)
Chronic pulmonary disease	7·20	0·007	0·55 (0·36, 0·85)
Depression, uncomplicated	6·84	0·009	1·83 (1·16, 2·88)
Metastatic cancer	4·65	0·031	0·67 (0·47, 0·96)
Weight loss	2·98	0·084	0·72 (0·50, 1·04)
Solid tumour without metastasis	2·23	0·135	1·50 (0·88, 2·56)
Hypothyroidism	0·70	0·404	1·21 (0·76, 1·92)
Renal failure	0·68	0·410	1·23 (0·75, 2·02)
Obesity	0·37	0·544	1·15 (0·72, 1·82)

Values in parentheses are 95 per cent confidence intervals. Note: Co‐variable effects listed in the table were estimated using separate multivariable weighted hierarchical generalized linear models for each study population. Results from each model are adjusted for patient age, for categories of co‐morbid disease that occurred in 5 per cent or more of the patient population and for the linear effect of hospital case volume.

## Discussion

This analysis of US hospital discharge data for procedures performed in 2012 indicates that hospital case volumes for pancreaticoduodenectomy, major hepatectomy and total gastrectomy were not statistically significant predictors of inpatient mortality, unlike patient age and co‐morbidity, which were highly significant predictors, for each of the assessed study populations.

Increased centralization of complex patient care has been an objective of both academic surgery and health policy initiatives for several decades^32^ ^33^. The present finding that there is no statistically significant relationship between hospital case volume and mortality risk in the current era may indicate that these efforts have been successful. Recent investigations^34^ ^35^ suggest that observed improvements over time in patient‐specific outcomes after pancreatic resection are associated with both patient migration to higher‐volume centres and reduced overall morbidity and mortality in lower‐volume centres.

Another explanation for these findings is that they reflect differences in the methods used to assess the effect of case volume. Many studies assessing the effect of hospital volume on mortality have used statistical methods that do not correct for clustering^9^, ^10^, ^11^, ^12^.

These findings may also be attributable in part to differences in the number and type of concurrent adjustments made for patient‐level co‐variables that contribute to mortality risk. Previous studies vary meaningfully in the adjustments made for patient co‐morbidity and other specific factors known to be associated with short‐term mortality risk^13^. The representation of the effect of volume also varies across studies. Where to set the thresholds for defining low volume is unclear. In the landmark 2002 study^1^, volume was categorized in quintiles. For example, annual pancreatectomy case volume was classified as very low volume (fewer than 1), low volume (1 or 2), medium volume (3–5), high volume (6–16) and very high volume (more than 16). Subsequent volume standards for pancreatic resection suggested by the Leapfrog group were set at 11 per year^36^. In the present study, 21·1 per cent of hospitals performed fewer than 11 pancreaticoduodenectomies per year.

A systematic review^7^ demonstrated wide variation in the terms used to define categories of hospital case volume for pancreaticoduodenectomy, with definitions of low volume ranging from fewer than one to fewer than 21 patients. Another systematic review^37^ described the same problem with liver resection volume levels, with low hospital case volume definitions ranging between fewer than two and fewer than 33 cases. Nearly 50 per cent of hospitals in the present study performed fewer than 11 major hepatectomies per year.

Study population definitions also vary across studies. Approximately one‐third of pancreatectomy volumes in the USA comprises patients who have distal pancreatectomy rather than pancreaticoduodenectomy, and these patients have a significantly lower risk of morbidity and mortality^38^. The present analysis was limited to patients with highest expected perioperative mortality rate, including only patients with complex HPB resections and total gastrectomy.

Relationships between hospital volume and cost of care have been under scrutiny recently. Some studies^39^ suggest higher case mix‐adjusted Medicare episode payments for selected complex cardiovascular operations performed at low‐volume centres, whereas others^40^ ^41^ have failed to identify any meaningful associations between risk‐adjusted payments and hospital volume in patients after cancer resections or liver surgery. Of interest, in both studies^40^ ^41^ postoperative complications were not associated with patient volume, but were associated with higher costs.

The present study has several important limitations. It assessed only one patient outcome, inpatient mortality. Results for this outcome are potentially biased by unmeasured differences among hospital discharge practices or other characteristics. Hospital case volume for complex HPB and upper GI resections may be related significantly to patient outcomes not assessed in the study, including 30‐ or 90‐day mortality, operative complications, duration of hospital stay, discharge to nursing facility, longer‐term survival and measures of oncological efficacy. Study populations were defined exclusively using the principal operation performed. Important differences may occur among hospitals with regard to other patient selection criteria, including features of the preoperative diagnosis and technical complexity. This study did not include many patient factors that are important in practice when considering outcomes for complex intra‐abdominal operations. These include the identification of aborted resections after initial exploration due to real or perceived technical unresectability, factors considered during preoperative consultation, and decision‐making criteria used to pursue surgery as a treatment option.

In this study, available population data and updated statistical methods were used to assess the effect of hospital case volume for GI and hepatobiliary operations on in‐hospital mortality, accounting for the confounding effects of patient‐level differences in co‐morbid disease. The effect of hospital case volume was evaluated as a linear function, as dichotomous categories above or below the median, and as a non‐linear continuous function, in three separate study populations. The results were the same for each assessment made. Patient co‐morbidity and not hospital case volume was a statistically significant predictor of inpatient mortality, for any study population, for any functional format used to represent the effect of hospital case volume on patent‐level mortality risk.
